# VNN1 as a potential biomarker for sepsis diagnosis and its implications in immune infiltration and tumor prognosis

**DOI:** 10.3389/fmed.2023.1236484

**Published:** 2023-08-07

**Authors:** Wei Guan, Jiaruo Xu, Yinghan Shi, Xiuli Wang, Shaoyan Gu, Lixin Xie

**Affiliations:** ^1^College of Pulmonary and Critical Care Medicine, Chinese PLA General Hospital, Beijing, China; ^2^Chinese PLA Medical School, Beijing, China

**Keywords:** sepsis, biomarkers, immune status, tumor prognosis, gene expression

## Abstract

**Background:**

This study explored novel biomarkers for diagnosing sepsis, a severe disease prevalent in clinical settings, particularly threatening to elderly patients.

**Methods:**

Using microarray gene expression datasets and fatty acid metabolism signatures, we identified differentially expressed genes between sepsis and healthy control groups. Correlations between candidate genes, immune cells, and immune function were assessed. Logistic regression analysis and single-gene GSEA analysis were performed to identify potential biomarkers. The biomarkers’ association with different types of tumors was investigated.

**Results:**

Twelve genes related to fatty acid metabolism were excluded. CA4, OLAN, and VNN1 were found relevant to immune cells and function. Among these, only VNN1 showed statistical significance (*p* < 0.05), with a strong area under the ROC curve (0.995). High VNN1 expression indicated activation of certain metabolic pathways, while low expression suggested potential autoimmune responses. VNN1 was up-regulated in eight tumors and down-regulated in eight others. High VNN1 expression was linked to poor prognosis in six types of tumors, and low expression was linked to poor prognosis in four types of tumors. VNN1 expression showed correlations with stromal scores, immune scores, and cancer purity in different types of tumors.

**Conclusion:**

VNN1 holds promise as a potential biomarker for sepsis diagnosis and is significant in identifying immune infiltration in tumor tissue and predicting tumor prognosis.

## 1. Introduction

Sepsis is a clinical disease that can be life-threatening. It occurs mainly in people with low immunity, such as the elderly, infants, people with underlying diseases, or people who use immunosuppressive drugs. Today, there are many types of antibiotic available to treat sepsis. However, the mortality rate from sepsis is still about 30%. Mortality may be higher when sepsis progresses to septic shock (1). Because it is not a specific disease, sepsis is considered a syndrome with uncertain pathophysiological characteristics (2). It can be identified by the clinical results of the suspected infected patient, such as routine blood tests, high-sensitivity C-reactive protein, procalcitonin, blood culture, and next generation sequencing (NGS). But there are still some limitations, such as interference in the identification of other non-infectious diseases, the time waiting for cultural outcomes and the cost of testing.

Fatty acid metabolism is necessary for the human body. It can produce triphosadenine (ATP) for cell consumption and is stored in living organisms as triglycerides. All cell membranes are made of fatty acids. Such as the nucleus, mitochondria, the endoplasmic reticulum, and the Golgi. In recent years, many studies have demonstrated the correlation between fatty acid metabolism and a variety of tumor diseases (3–6). However, the mechanism of fatty acid metabolism in sepsis remains need to be explored. The innate and adaptive immune systems play a key role in the host response to sepsis (7). There have been many investigations that have found a role for immunomodulatory therapies in improving the long-term prognosis of patients with sepsis (8).

Because there is no golden standard for diagnosing sepsis in clinical settings, we want to look for potential biomarkers based on fatty acid metabolism and further investigate the correlations between these genes and immune cells (IMC) and immune function (IMF). We wanted to further explore the correlations between these biomarkers and different types of tumors.

## 2. Methods

### 2.1. Acquisition of sepsis microarray data sets and genes associated with fatty acid metabolism

The sepsis microarray data sets were selected and downloaded from the Gene Expression Omnibus database.^1^ GSE134347 (whole blood, 156 sepsis samples, and 83 healthy control samples) was set as primary and training data. GSE69063 (peripheral blood, 57 sepsis samples and 11 healthy control samples) and GSE54514 (whole blood, 35 sepsis samples and 18 healthy control samples) were established as supplement and validation data. Then we downloaded the pan-cancer data set from the UCSC^2^ database: TCGA Pan-cancer (PANCAN, *N* = 10,535, *G* = 60,499), and extracted expression data of the VNN1(ENSG00000112299) gene in each sample, carried out a log2 (x + 0.001) transformation, and finally we also eliminated cancer types with fewer than 3 samples in a single cancer type, and finally obtained the expression data of 26 cancer types.

We downloaded all gene sets from the Molecular Signature Database (MSigDB, version 7.5.1).^3^ After exploring it, we found that there were three sets containing fatty acid metabolism genes (Kyoto Encyclopedia of Genes and Genomes (KEGG) fatty acid metabolism pathways; 42 genes, Hallmark fatty acid metabolism genes; 177 genes, and Reactome fatty acid metabolism genes;158 genes). After removing duplicate genes, we obtained 309 genes related to fatty acid metabolism. The general idea and data processing are shown in the flow chart.

### 2.2. Identification of different expression genes in fatty acid metabolism genes

Differential expression genes of the GSE134347 data set were calculated using the ‘limma’ R package (9) between sepsis and healthy control group. we selected the value of the mean probe as the gene expression value if there were multiple probes for one gene and then chose the genes with the following threshold: adjust the *p* < 0.05 and | Log2FC | (fold change) > 1.0. After intersecting with fatty acid metabolism genes, we obtained eligible FAMGs for further research.

### 2.3. Immune microenvironment and correlation with DEG fatty acid metabolism genes

The ‘ssGSEA’ function implemented in the ‘GSVA’ package R (10) was used to evaluate Immune Cells (IMC) and immune function (IMF) of the GSE134347 dataset (sepsis group versus healthy group). Subsequently, we evaluated the correlations between FAMGs and the immune microenvironment. The most relevant genes with IMC and IMF were considered potential biomarkers.

### 2.4. Validation of the receiver operating characteristic curve and single gene GSEA analysis

Logistic regression was used to further screen for potential biomarkers (*p* < 0.05). The receiver operating characteristic curve (ROC) was used to evaluate its diagnostic accuracy. The sepsis group of the GSE134347 dataset was divided into two groups according to the mean expression of biomarkers. Single-gene GSEA analysis was used to observe different activated pathways between the high expression group and the low expression group. The GSE69063 dataset was used to search for differences in biomarker expression between the sepsis group and the healthy group and further explored whether there were differences in emergency departments (T0), 1 h later (T1) and 3 h after arrival (T2). The GSE54514 dataset was used to validate the different activated pathways between the high-expression group of biomarkers and the low group.

### 2.5. Pan-cancer analysis

We used R software (version 4.1.3) to calculate the expression difference of VNN1 between normal samples and tumor samples in each tumor. The “Coxph” function of R package “survival” (3.4.0) was used to calculate the correlations between the overtime survival of each tumor and the expression level of VNN1. The R package “ESTIMATE” (version 1.0.13) was used to calculate the stromal, immune, and estimate scores in each sample of different type of tumors based on gene expression. Finally, we obtained the immune infiltration scores of 9,555 tumor samples in a total of 39 tumor types. We used the corr.test function of the R package “psych” (version 2.2.5) to calculate the Pearson’s correlation coefficient between VNN1 gene expression and immune infiltration scores in each tumor, to determine the significantly correlated immune infiltration score. Finally, we integrated the purity data of tumor samples and VNN1 gene expression data for correlation analysis.

### 2.6. Statistical analysis

All statistical analyzes in this paper were performed on R software (version 4.1.3). Linear fitting and empirical Bayes implanted in the ‘limma’ package R were used to search for differential expression genes between the sepsis group and the healthy control group. Pearson’s correlation analysis was applied to find correlations between genes and immune cells and immune function. It was also used to find the correlations between expression of VNN1, immune infiltration scores and the purity of different type of tumors. The Wilcox test was used to estimate differences in immune status in the GSE134347 data set and different expressions of VNN1 in the GSE69063 data set between the sepsis group and the healthy control group. All *p* < 0.05 (bilateral) was considered statistically significant. The logarithmic ranking test was used to obtain the tumor prognostic significance. Unpaired Student’s *t*-test for significant difference analysis between pairs. The unpaired Wilcoxon rank sum and Signed Rank Tests was used to explore the significance of the difference between normal and tumor samples in each tumor type.

## 3. Results

### 3.1. Identification of DEGs of fatty acid metabolism between sepsis and healthy control

**Figure 1:**
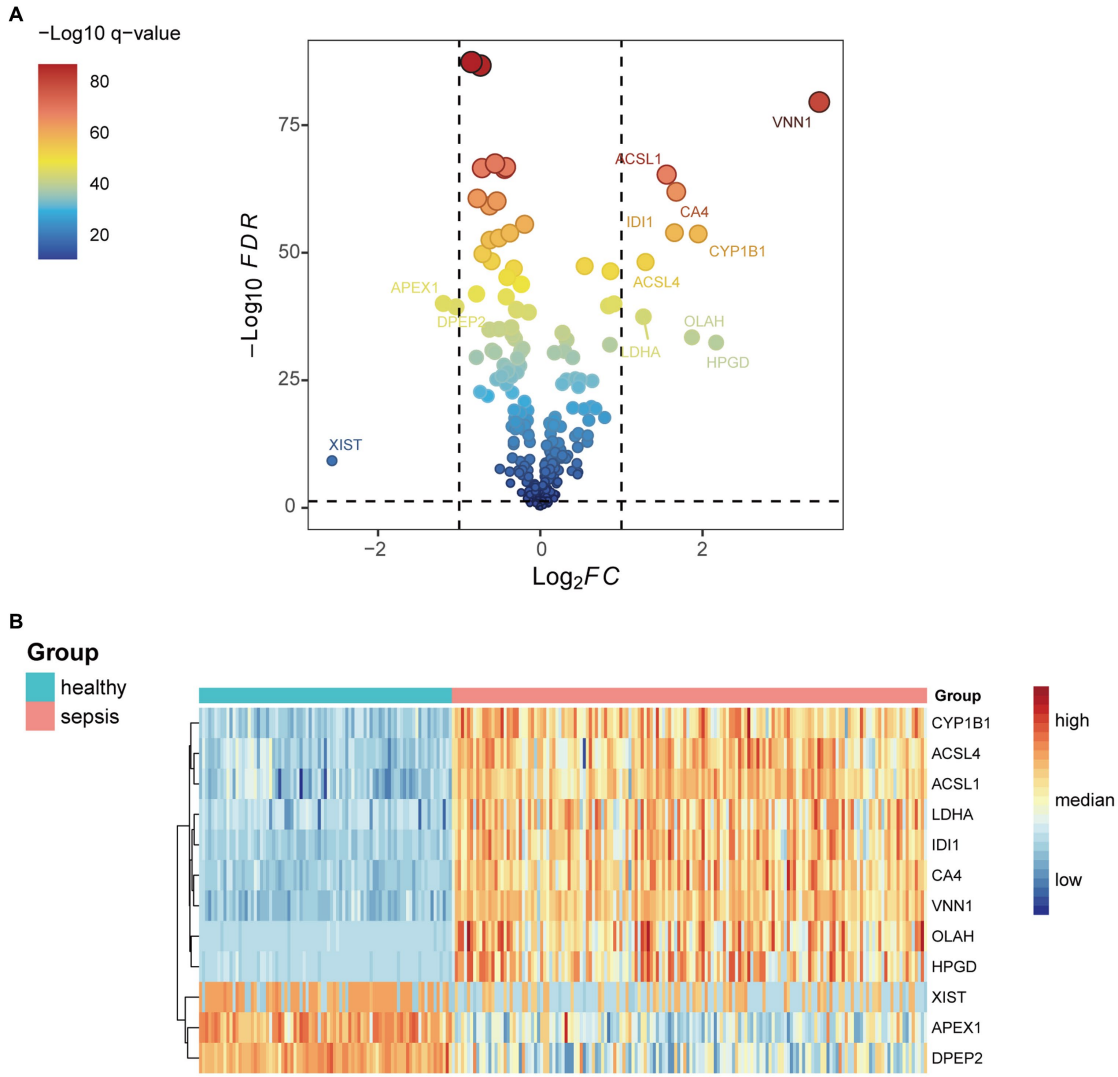
**(A)** Differential genes related to fatty acid metabolism in the GSE134347 data set. **(B)** Expressions of the 12 most differential genes between sepsis and healthy groups in GSE134347 data set.

**Figure 2:**
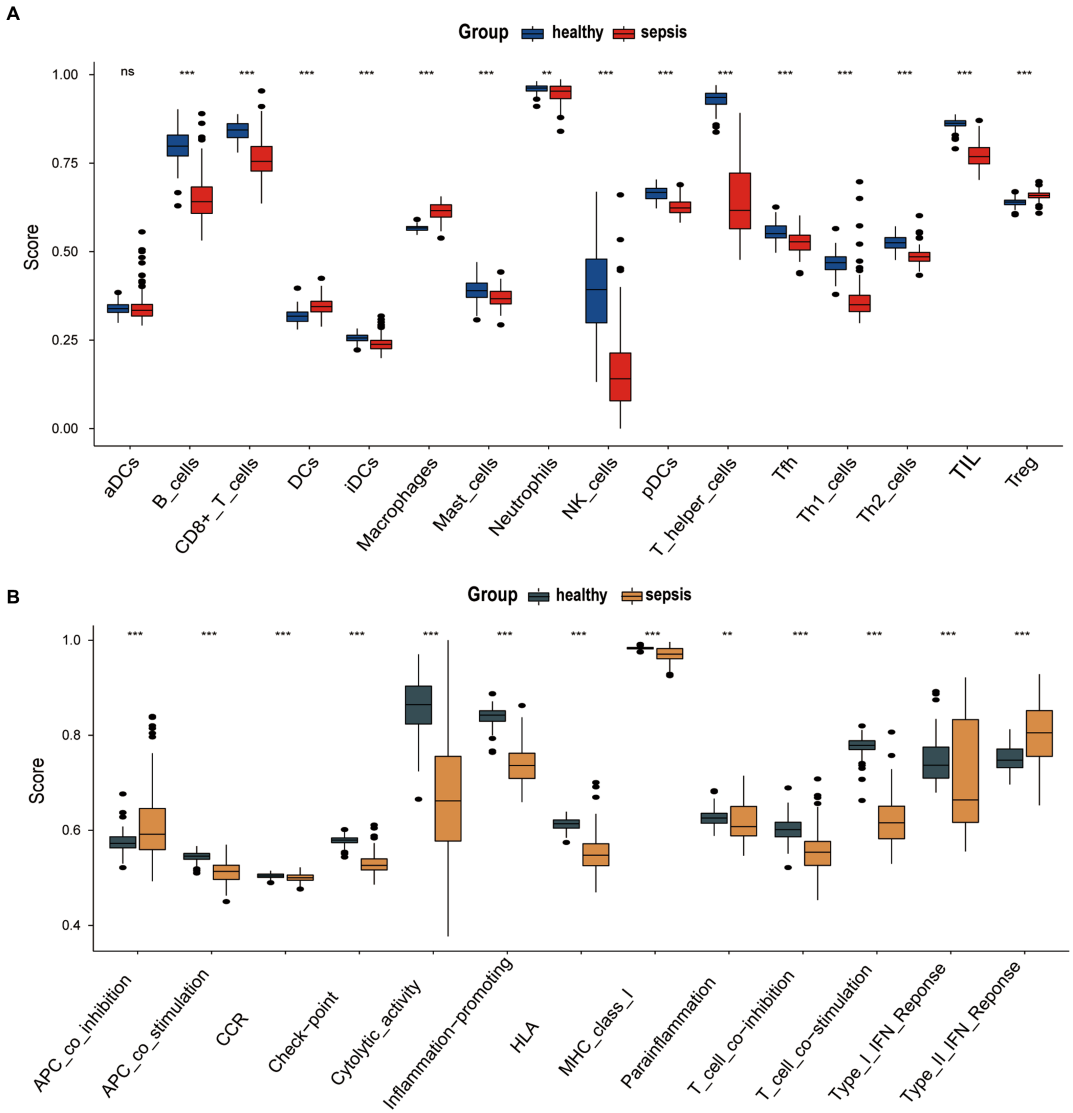
**(A)** Differences infiltration of immune cell markers (IMC) between sepsis group and the healthy group of GSE134347. **(B)** Differences immune function markers (IMF) markers between sepsis group and the healthy group of GSE134347.

Differential expression genes in the GSE134347 data set between the sepsis group and the healthy control group were calculated using the ‘limma’ R package. After intersecting with fatty acid metabolism genes, we obtained their differential expression. According to the set threshold (adjust *p* < 0.05 and |Log2FC| > 1.0), 12 genes (Figure 1) were screened out as DEGs of fatty acid metabolism. As shown in Figure 2, we found that ACSL4, CYP1B1, ACSL1, OLAH, HPGD, VNN1, CA4, LDHA, and IDI1 were high expression in the sepsis group. In contrast, APEX1, XIST, and DPEP2 were high expression in the healthy control group.

### 3.2. Immune microenvironment

**Figure 3:**
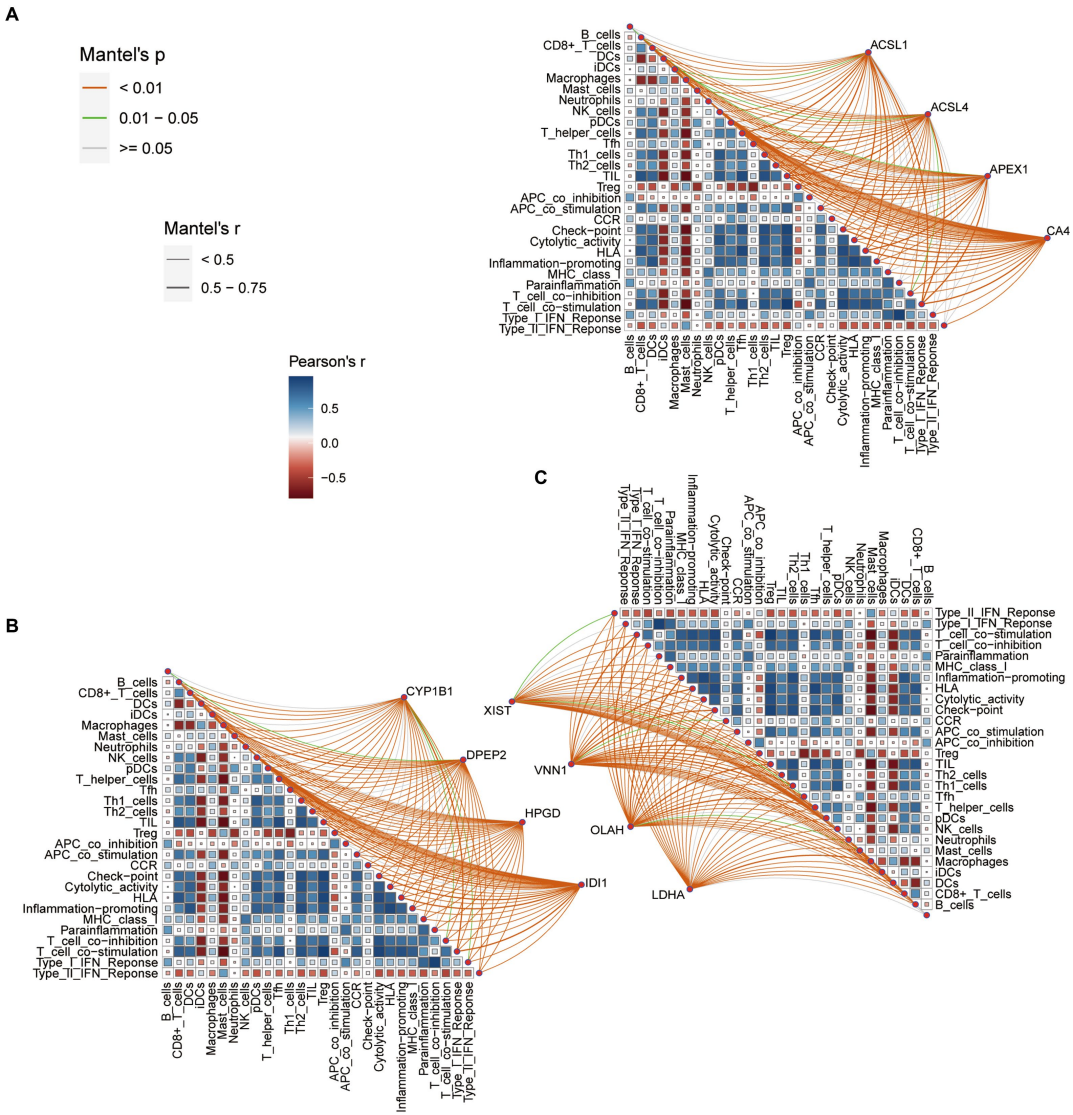
**(A)** Correlation between the fatty acid metabolism (FAM) genes (ACSL1, ACSL4, APEX1 and CA4), immune cell markers (IMC) and immune function markers (IMF). **(B)** Correlation between the fatty acid metabolism (FAM) genes (CYP1B1, DPEP2, HPGD and IDI1), immune cell markers (IMC) and immune function markers (IMF). **(C)** Correlation between the fatty acid metabolism (FAM) genes (XIST, VNN1, OLAH and LDHA), immune cell markers (IMC) and immune function markers (IMF).

The ‘GSVA’ R package was used to assess the immune microenvironment of the GSE134347 data set. We analyze the differences in infiltration of immune cell markers (IMC) and immune function markers (IMF) markers between sepsis group and the healthy control group using the ssGSEA algorithm. Figure 3A shows the differential expression of 16 immune cell markers in two groups. Dendritic cells (DCs), Macrophages, and T cells regulatory (Treg) have a significantly higher expression in the sepsis group. Twelve immune cell markers [B cells, CD8+ T cells, immature Dendritic Cells (iDCs), Mast cells, Neutrophils, NK cells, plasmacytoid Dendritic Cells (pDCs), T helper cells, T follicular helper cells, Type 1 T- helper cells, Type 2 T-helper cells, Tumor-Infiltrating Lymphocyte (TIL)] have a lower expression in the healthy control group. However, there was no significant difference in the expression of activated dendritic cells (aDC) between two groups. There are 13 Immune Functions (IMF) markers shown in Figure 3B, APC co-inhibition, and Type II IFN response have higher expression in the sepsis group. Meanwhile, 11 types of IMF (APC co-stimulation, Chemokine Receptor (CCR), checkpoint, cytolytic-activity, Human Leukocyte Antigen (HLA), inflammation-promoting, Major Histocompatibility Complex (MHC) class I, Para-inflammation, T cell co-inhibition, T cell co-stimulation, Type I IFN response) have high expression in the healthy control group.

### 3.3. Correlations between fatty acid metabolism DEGs and IMC and IMF

Figures 3A–C shows the correlation between the fatty acid metabolism DEGs, IMF and IMC. We set the threshold value as follows: adjusted *p* < 0.001, |*r*| > 0.75. According to this setting, we discover that VNN1 was negatively correlated with checkpoint, HLA, inflammation-promoting, T cell co-stimulation, T helper cells, TIL, and positively correlated with macrophages. The OLAH was negative with CD8+ T cells, HLA, inflammation-promoting, T cell co-stimulation, T helper cells, Th1 cells, and TIL.

The CA4 was negatively correlated with checkpoint, HLA, T cell co-stimulation, T helper cells, Th1 cells, TIL, and positively correlated with macrophages. The above three genes were associated with seven IMF or IMC phenotypes, which were eligible for further study. However, the remaining nine DEGs of the fatty acid metabolism were excluded because they had fewer relevant numbers with IMC and IMF.

### 3.4. Logistic regression and receiver operating characteristic curve validation

**Figure 4:**
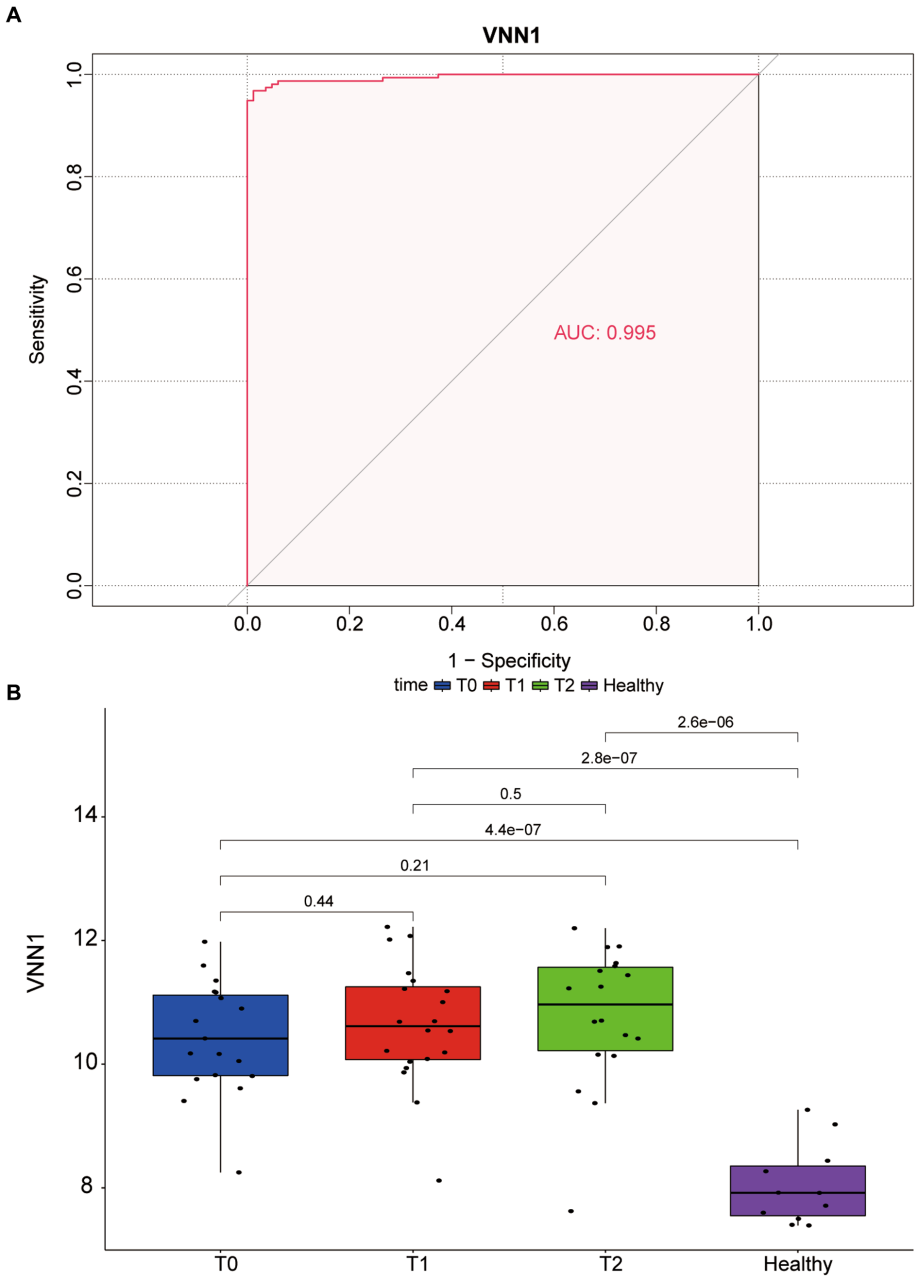
**(A)** The area under receiver operating characteristic (ROC) curve of VNN1 in GSE134347. **(B)** Diverse expression of VNN1 in 4 groups of GSE69063 datasets. T0: patients on arrival in the emergency. T1: 1 h after the patient arrives at the emergency room; T2: 3 h after the patient arrives at the emergency room. Healthy: healthy control group.

Logistic regression was used to assess the likelihood of sepsis based on CA4, OLAH, and VNN1. As shown in Table 1, only the *p-value* of VNN1 was less than 0.05. Figure 4A shows the area under receiver operating characteristic curve of VNN1 (AUC value: 0.995) in the GSE134347 data set. To make the outcome persuasive, we chose the GSE69063 data set to validate the diverse expression of VNN1 between the healthy control group and sepsis, including patients on arrival in the emergency departments (T0), 1 h later, and 3 h after arrival (Figure 4B). The expression of VNN1 in the healthy group was significantly lower than in the sepsis group. But there were no differences between the T0, T1, and T3 groups.

**Table 1:** Results of logistic regression for CA4, OLAH and VNN1.

Gene	Estimate std.	Error	*z*-value	*p*-value
CA4	1.346	1.742	0.772	0.43993
OLAH	5.060	4.256	1.189	0.23456
VNN1	2.541	1.169	2.174	0.02967

### 3.5. Single-gene GSEA-KEGG analysis

**Figure 5:**
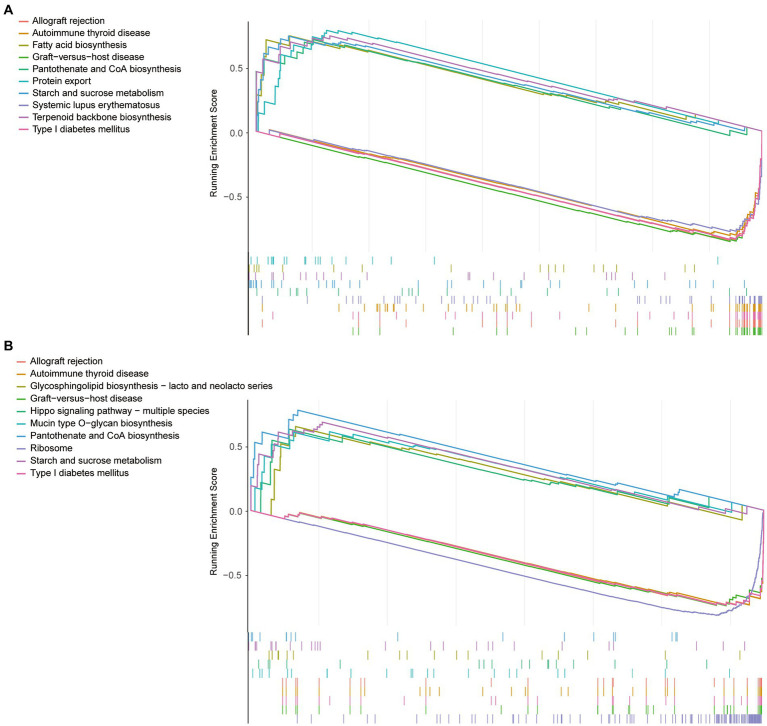
**(A)** The top10 pathways enriched based on VNN1 expression of GSE134347. **(B)** The top10 pathways enriched based on VNN1 expression of GSE54514.

To observe differences in pathway activation, the sepsis samples in the GSE134347 dataset were divided into two groups according to the mean expression of VNN1 and sepsis samples in the GSE54514 dataset were processed in the same way. The top10 pathways enriched based on VNN1 expression (5 in high expression and 5 in low expression) were illustrated in Figure 5 (Figure 5A: GSE134347, Figure 5B: GSE54514). After a comprehensive comparison, we found that the genes in the high expression group were mainly enriched in protein export, fatty acid biosynthesis, terpenoid backbone biosynthesis, starch and sucrose metabolism, pantothenate and CoA biosynthesis. Meanwhile, genes in the low expression group were mainly enriched in graft-versus-host disease, allograft rejection, type I diabetes mellitus, autoimmune thyroid disease, and systemic lupus erythematosus. In GSE54514, we observed that genes in high expression of VNN1 were mainly enriched in pantothenate and CoA biosynthesis, starch and sucrose metabolism, glycosphingolipid biosynthesis, hippo signaling pathway, mucin type O-glycan biosynthesis. On the genes in low expression group of VNN1 were mainly enriched in ribosome, graft-versus-host disease, type I diabetes mellitus, autoimmune thyroid disease, and allograft rejection. We found that starch and sucrose metabolism, pantothenate and CoA biosynthesis were both highly activated in the high expression group of VNN1 in GSE134347 and GSE54514 datasets. Pathways of graft-versus-host disease, type-I diabetes mellitus, autoimmune thyroid disease, and allograft rejection were both highly activated in the low expression group of these two datasets.

### 3.6. Pan-cancer analysis

**Figure 6:**
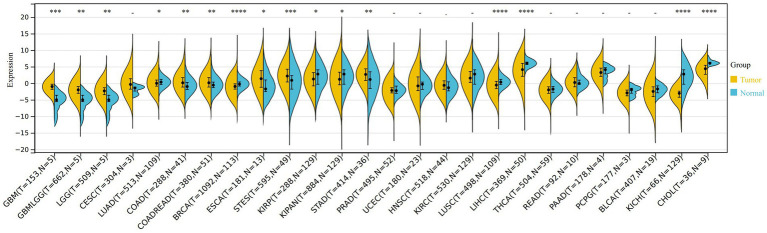
The different expression of VNN1 in 26 types of tumors between normal samples and tumor samples. *0.05, **0.01, ***0.001, ****0. 0001. Yellow: tumor group, Blue: normal group.

After comparing the expression of VNN1 in tumor tissues and paired normal tissues, we found that there were significant differences among 16 types of tumors, among which 8 types of tumors tissues [GBM (tumor: −1.08 ± 1.27, normal: −5.53 ± 2.64, *p* = 2.3e-4), GBMLGG (tumor: −2.13 ± 1.74, normal: −5.53 ± 2.64, *p* = 1.6e-3), LGG (tumor: −2.44 ± 1.74, normal: −5.53 ± 2.64, *p* = 2.9e-3), COAD (tumor: 0.47 ± 2.24, normal: −0.61 ± 1.67, *p* = 3.3e-3), COADREAD (tumor: 0.42 ± 2.15, normal: −0.40 ± 1.71, *p* = 9.1e-3), ESCA (tumor: 1.53 ± 3.14, normal: −0.64 ± 2.98, *p* = 0.01), STES (tumor: 2.30 ± 2.73, normal: 0.51 ± 3.55, *p* = 2.4e-4), STAD (tumor: 2.64 ± 2.45, normal: 0.92 ± 3.68, *p* = 2.9e-3)] were significantly up-regulated, while the other 8 types of tumors tissues [LUAD (tumor: 0.19 ± 1.85, normal: 0.37 ± 1.09, *p* = 0.03), BRCA (tumor: −0.95 ± 1.36, normal: −0.23 ± 1.06, *p* = 2.1e-10), KIRP (tumor: 1.43 ± 2.91, normal: 1.90 ± 2.65, *p* = 0.04), KIPAN (tumor: 1.38 ± 2.94, normal: 1.90 ± 2.65, *p* = 0.02), LUSC (tumor: −0.44 ± 1.90, normal: 0.37 ± 1.09, *p* = 5.5e-8), LIHC (tumor: 3.72 ± 2.89, normal: 6.02 ± 0.80, *p* = 3.8e-9), KICH (tumor: −3.51 ± 1.96, normal: 1.90 ± 2.65, *p* = 3.6e-25), CHOL (tumor: 3.92 ± 2.03, normal: 6.24 ± 0.61, *p* = 4.9e-5)] were significantly down-regulated (Figure 6).

**Figure 7:**
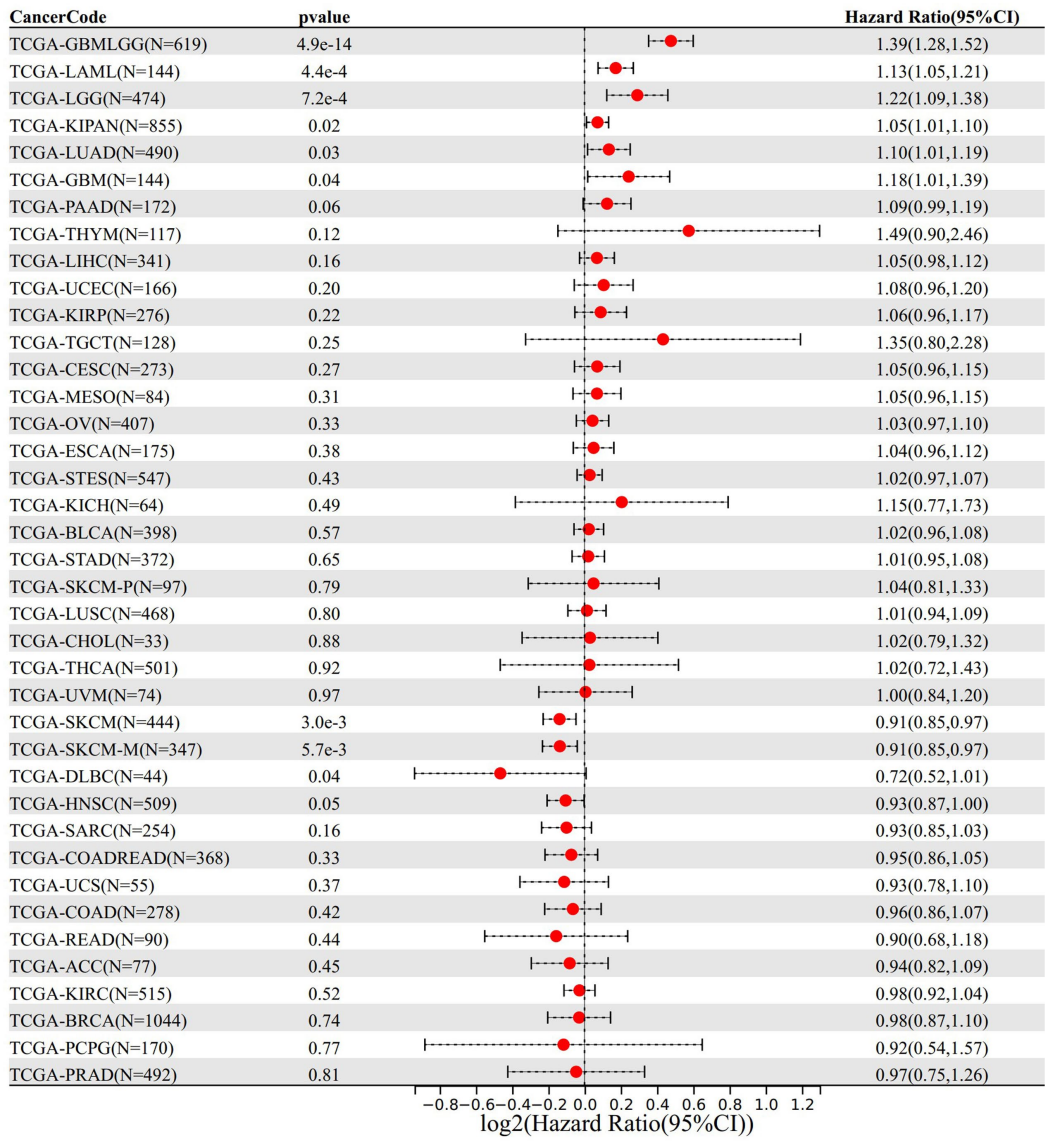
The relationship between VNN 1 gene expression and prognosis in 39 types of tumors.

In the analysis of gene expression and overall survival time, we found that high expression of VNN1 in 6 tumor types [TCGA-GBMLGG (*N* = 619, *p* = 4.9e-14, HR = 1.39 (1.28, 1.52)), TCGA-LGG (*N* = 474, *p* = 7.2e-4, HR = 1.22 (1.09, 1.38)), TCGA-LUAD (*N* = 490, *p* = 0.03, HR = 1.10 (1.01, 1.19)), TCGA-LAML (*N* = 144, *p* = 4.4e-4, HR = 1.13 (1.05, 1.21)), TCGA-KIPAN (*N* = 855, *p* = 0.02, HR = 1.05 (1.01, 1.10)), TCGA-GBM (*N* = 144, *p* = 0.04, HR = 1.18 (1.01, 1.39))] had poor prognosis, and low expression of VNN1 in 4 tumor types [TCGA-HNSC (*N* = 509, *p* = 0.05, HR = 0.93 (0.87, 1.00)), TCGA-SKCM-M (*N* = 347, *p* = 5.7e-3, HR = 0.91 (0.85, 0.97)), TCGA-SKCM (*N* = 444, *p* = 3.0e-3, HR = 0.91 (0.85, 0.97)), TCGA-DLBC (*N* = 44, *p* = 0.04, HR = 0.72 (0.52, 1.01))] had poor prognosis (Figure 7).

The immune infiltration analysis mainly includes three scoring indicators (Stromal Score, Immune Score and ESTIMATE Score), we calculated the above three scoring methods on 26 different types of tumors and searched for the correlations between these three scoring results and the expression of VNN1 gene. In Stromal Score analysis, we found that in 6 types of tumors [GBM (*r* = 0.674, *p* = 1.68e-21), GBMLGG (*r* = 0.669, *p* = 2.65e-86), SARC (*r* = 0.661, *p* = 1.00e-33), KICH (*r* = 0.647, *p* = 5.66e-09), LGG (*r* = 0.616, *p* = 4.42e-54) and SKCM-M (*r* = 0.602, *p* = 5.34e-36), threshold value: *p* < 0.05, *r* > 0.60] have positively correlations with the expression of VNN1. We also found that in Immune Score and Estimate score analysis, there were 6 types of tumors [GBMLGG (*r* = 0.704, *p* = 2.03e-99), TGCT (*r* = 0.704, *p* = 4.77e-21), KICH (*r* = 0.694, *p* = 1.44e-10), GBM (*r* = 0.687, *p* = 1.42e-22), SARC (*r* = 0.683, *p* = 8.58e-37), LGG (*r* = 0.660, *p* = 2.66e-64), threshold value: *p* < 0.05, *r* > 0.65] and 5 types of tumors [TCGT (*r* = 0.724, *p* = 1.00e-22), SARC (*r* = 0.724 *p* = 3.84e-43), GBMLGG (*r* = 0.708 *p* = 8.33e-101), GBM (*r* = 0.708 *p* = 2.08e-24), KICH (*r* = 0.700, *p* = 8.57e-11), threshold value: *p* < 0.05, *r* > 0.70] have positively correlations with the expression of VNN1, respectively (Figures 8A–C).

**Figure 8:**
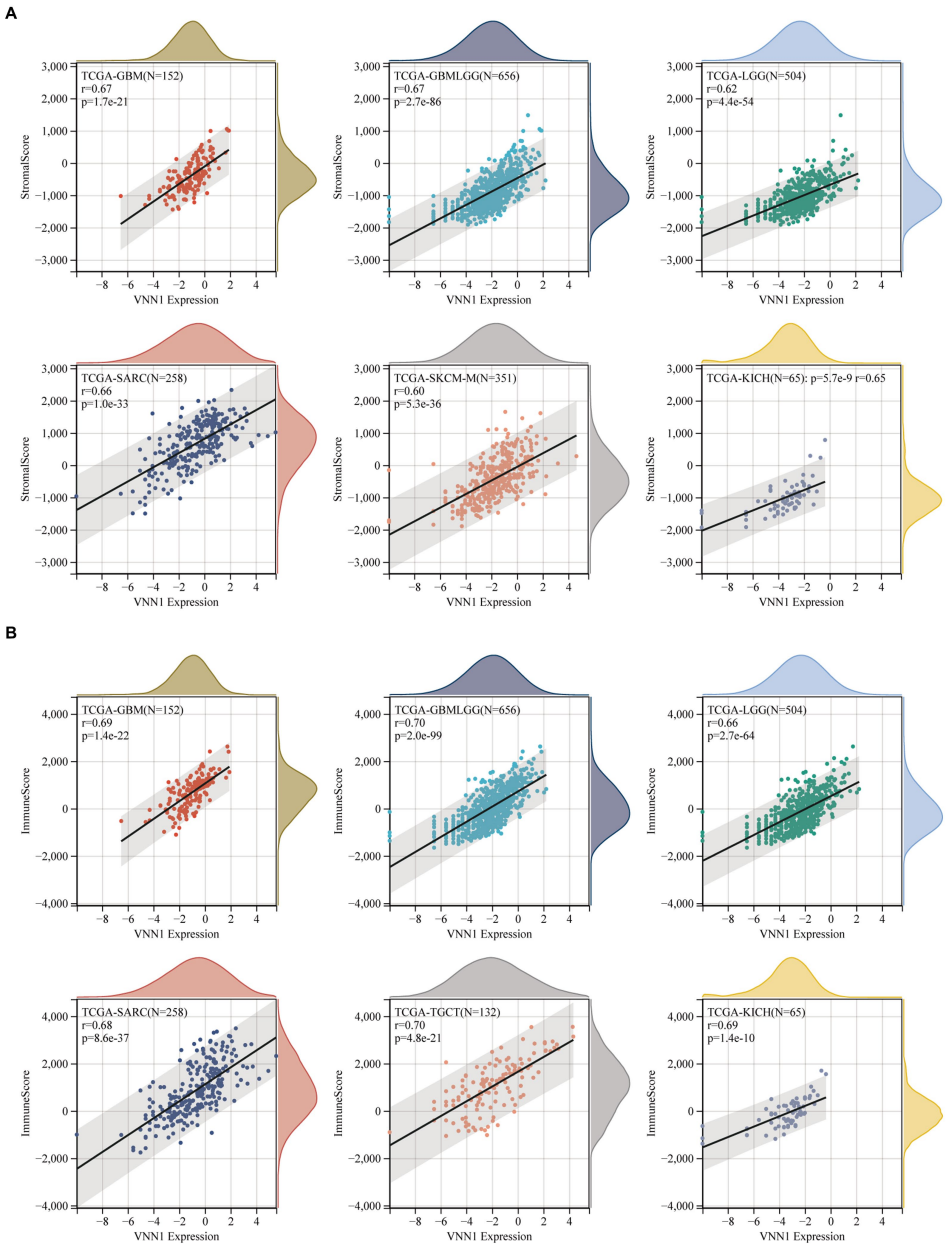


**Figure 8:**
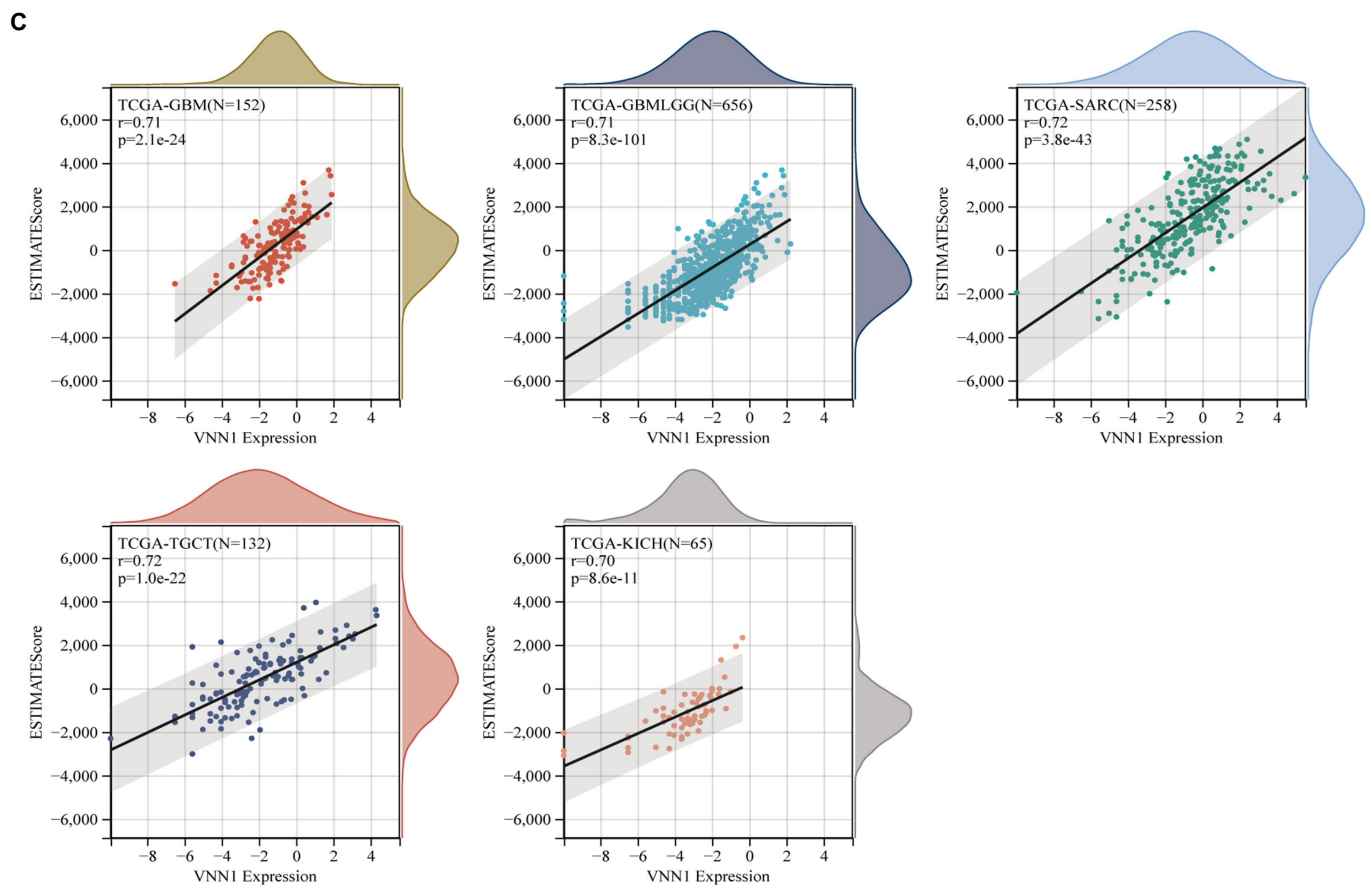
**(A)** The correlations between the expression and stromal score. **(B)** The correlations between the expression and immune score. **(C)** The correlations between the expression and estimate score.

**Figure 9:**
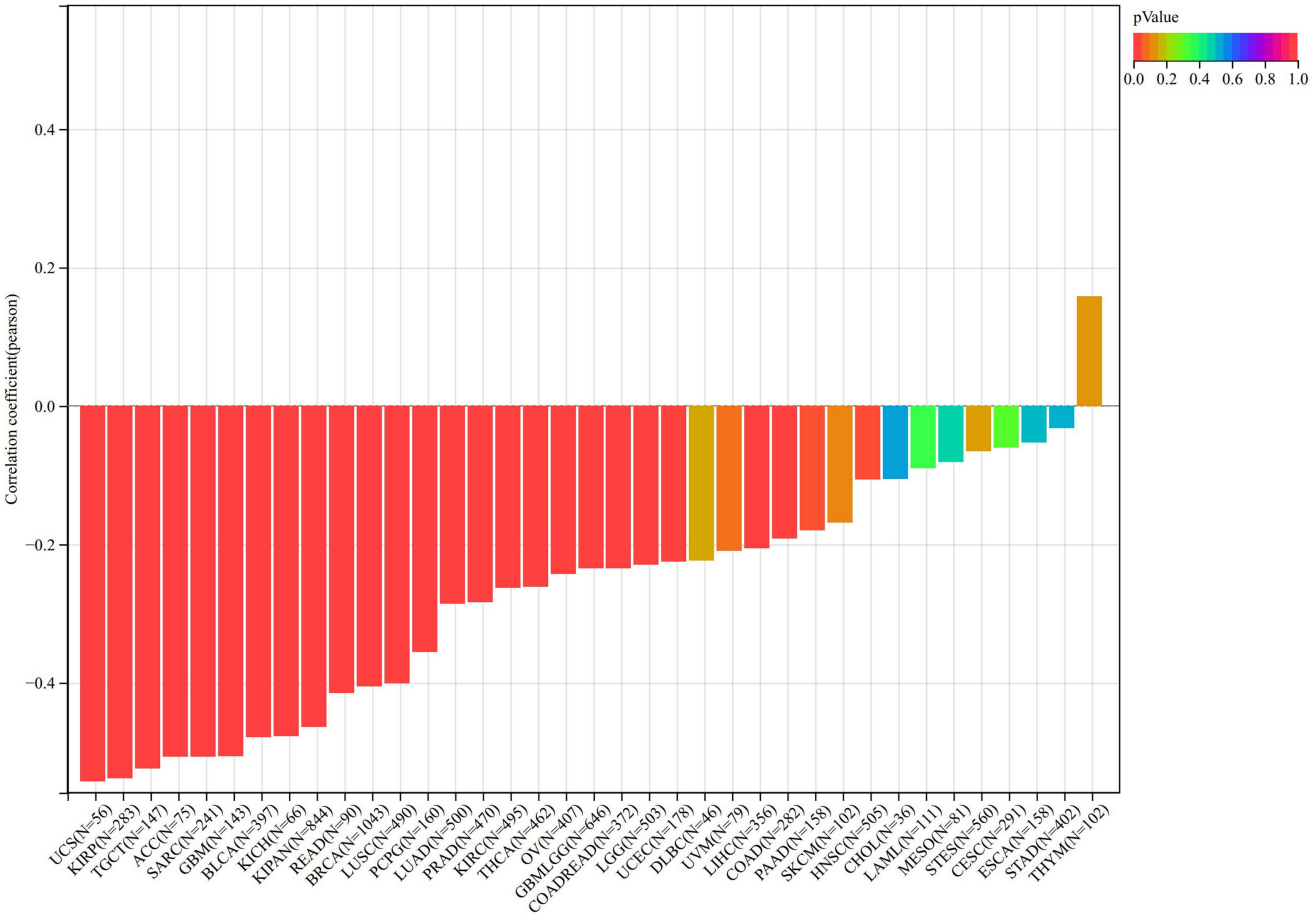
The correlation between the expression of VNN1 and the purity of 26 types of tumors.

Finally, we observed a negative correlation between the purity of 6 different types of tumors [UCS (*r* = −0.543, *p* = 1.55e-05), KIRP (*r* = −0.538, *p* = 1.25e-22), TGCT (*r* = −0.524, *p* = 9.49e-12), ACC (*r* = −0.507, *p* = 3.46e-06), SARC (*r* = −0.507, *p* = 3.89e-17), GBM (*r* = −0.506, *p* = 1.13e-10)] and the expression of VNN1 (Figure 9).

## 4. Discussion

Sepsis is a clinically critical disease caused by pathogenic microorganisms that invade the human body. It is a systemic inflammatory response syndrome and clinical manifestations include fever, chills, increased breathing and heart rate, concomitant fatigue, general muscle soreness, mental excitement, irritability, or lethargy. The pathophysiology that clinicians can observe mainly includes high oxygen consumption, hyperventilation, hyperglycemia, increased proteolysis, and hyperlactatemia, increased cardiac output, and decreased peripheral vascular resistance. Early and prompt diagnosis and treatment can reduce mortality and reduce the likelihood of conversion to severe sepsis and septic shock. Few studies on the correlation between sepsis and signatures of fatty acid metabolism. The purpose of this study is to explore the immune status and relationship between sepsis and genes related to fatty acid metabolism through multiple GEO datasets and to further screen potential biomarkers for the diagnosis of sepsis.

Immune dysfunction occurred in both innate and adaptive immunity in patients with sepsis (11). Our research shows that dendritic cells, macrophages, and T cells regulatory were up regulated in the sepsis group. However, B-cells, Neutrophils, NK-cells, plasmacytoid Dendritic Cells, and immature Dendritic Cells were down-regulated in sepsis. Due to the induction of caspase 3-dependent apoptosis, the DCs in the spleen and lymph nodes may suffer profound loss (12). Other studies have also shown the simultaneous loss of immature dendritic cells in the abdominal cavity and splenic dendritic cells in cecal ligation and puncture-induced sepsis in mice (13). DCs activation of DCs can cause a rapid clustering of granulocytes, NK cells, and monocytes during a bacterial infection (14). However, paralyzed DCs may impair the function of activating innate immune cells and may promote the accumulation of the Tregs in organs to create an immunosuppressive environment by secreting a higher amount of TGF-β (15). That may also explain the up-regulate of APC co-inhibition in the sepsis group of our study.

The Vanin gene family has been identified in the human body, and the encoding protein includes three isoforms (VNN1, VNN2 and VNN3) (16). VNN1 is the main isoform of the VNN protein family in humans (17). As a precursor of CoA, pantothenic acid can be generated by pantetheinase metabolizing the pantetheine (18). Pantetheine is also the substrate of vanin-1 which improve vasculopathy in inflammatory conditions by protecting endothelial cells (19). Previous research has shown that the attenuated vasoprotective effects of pantetheine may be due to the overactivity in the vanin-1/pantetheinase pathway (20, 21). This is consistent with what we found that in two datasets (GSE134347 and GSE54514), the genes in the high expression were of VNN-1 group mainly enriched in pantothenate and CoA biosynthesis compared with the low expression group. The AUC value in the GSE134347 dataset can illustrate the importance of VNN1 in diagnosing sepsis and it can be validated in GSE69063 dataset by comparing the different expressions of VNN1 between the healthy group and sepsis group. The reason we did not find differences in T0, T1, and T3 groups may be the blood test interval is too short (within 3 h). Recently, some articles showed that inhibition of the VNN1 protein can alleviate the lung injury in sepsis and sepsis shock mice (22, 23).

Sepsis has been reported to be a common complication in immunosuppressed cancer patients and is associated with high morbidity and mortality (24). In the meantime, some previous studies have reported that the VNN1 gene is associated with the prognosis of certain tumors and tumor-related complications (25, 26). To further explore the association between sepsis and tumors in this study, based on VNN1, the difference expression between tumor and normal tissue, the overtime survival of tumor patients, the immune microenvironment of tumor tissue, and the purity of tumor tissue were analyzed using the method of pan-cancer analysis. However, the value of VNN1 in the diagnosis of tumors and the evaluation of the prognosis of tumor patients’ needs further clinical verification research.

## 5. Conclusion

In conclusion, the objective of our study is to search for potential biomarkers in sepsis based on fatty acid metabolism-related signatures, and finally VNN1 was screened. we further explored the differences in the immune microenvironment between the sepsis group and the healthy control group. In the end, we investigated the differences in activated pathways between the high expression group and the low expression group. This study searched for a potential biomarker of sepsis (VNN1), which will help to further explore the mechanism of action of fatty acid metabolism in sepsis, the immune response in sepsis, and the relationship between sepsis and tumors. However, there are still some limitations in this study. First, the different pathway regulation mechanisms between high expression and low expression of VNN1 need to be further researched. Second, the biomarker was screened from a public database and needed to be validated from more experimental verification.

## Data availability statement

The original contributions presented in the study are included in the article/supplementary material, further inquiries can be directed to the corresponding author.

## Author contributions

LX designed the study and revised the manuscript. WG, JX, YS, XW, and SG performed data analysis. WG drafted the manuscript. All authors contributed to the article and approved the submitted version.

## Funding

The study was supported by the China Key Scientific Grant Program (No. 2021YFC0122500).

## Conflict of interest

The authors declare that the research was conducted in the absence of any commercial or financial relationships that could be construed as a potential conflict of interest.

## Publisher’s note

All claims expressed in this article are solely those of the authors and do not necessarily represent those of their affiliated organizations, or those of the publisher, the editors and the reviewers. Any product that may be evaluated in this article, or claim that may be made by its manufacturer, is not guaranteed or endorsed by the publisher.

## Footnotes

^1^ https://www.ncbi.nlm.nih.gov/geo/

^2^ https://xenabrowser.net/

^3^ https://www.gsea-msigdb.org/gsea/msigdb

## References

[1] Huet O Chin-Dusting JP. Septic shock: desperately seeking treatment. Clin Sci (Lond). (2014) 126:31–9. doi: 10.1042/CS20120668, PMID: 24020445

[2] Singer M Deutschman CS Seymour CW Shankar-Hari M Annane D Bauer M. The third international consensus definitions for Sepsis and septic shock (Sepsis-3). JAMA. (2016) 315:801–10. doi: 10.1001/jama.2016.0287, PMID: 26903338 PMC4968574

[3] Currie E Schulze A Zechner R Walther TC Farese RV Jr. Cellular fatty acid metabolism and cancer. Cell Metab. (2013) 18:153–61. doi: 10.1016/j.cmet.2013.05.017, PMID: 23791484 PMC3742569

[4] Jin Z Chai YD Hu S. Fatty acid metabolism and Cancer. Adv Exp Med Biol. (2021) 1280:231–41. doi: 10.1007/978-3-030-51652-9_16 33791986

[5] Hoy AJ Nagarajan SR Butler LM. Tumour fatty acid metabolism in the context of therapy resistance and obesity. Nat Rev Cancer. (2021) 21:753–66. doi: 10.1038/s41568-021-00388-4, PMID: 34417571

[6] Corbet C Pinto A Martherus R Santiago de Jesus JP Polet F Feron O. Acidosis drives the reprogramming of fatty acid metabolism in Cancer cells through changes in mitochondrial and histone acetylation. Cell Metab. (2016) 24:311–23. doi: 10.1016/j.cmet.2016.07.003, PMID: 27508876

[7] Rimmelé T Payen D Cantaluppi V Marshall J Gomez H Gomez A. Immune cell phenotype and function in SEPSIS. Shock. (2016) 45:282–91. doi: 10.1097/SHK.0000000000000495 26529661 PMC4752878

[8] Delano MJ Ward PA. The immune system's role in sepsis progression, resolution, and long-term outcome. Immunol Rev. (2016) 274:330–53. doi: 10.1111/imr.12499, PMID: 27782333 PMC5111634

[9] Ritchie ME Phipson B Wu D Hu Y Law CW Shi W. Limma powers differential expression analyses for RNA-sequencing and microarray studies. Nucleic Acids Res. (2015) 43:e47. doi: 10.1093/nar/gkv007, PMID: 25605792 PMC4402510

[10] Hänzelmann S Castelo R Guinney J. GSVA: gene set variation analysis for microarray and RNA-seq data. BMC Bioinformatics. (2013) 14:7. doi: 10.1186/1471-2105-14-7, PMID: 23323831 PMC3618321

[11] van der Poll T Shankar-Hari M Wiersinga WJ. The immunology of sepsis. Immunity. (2021) 54:2450–64. doi: 10.1016/j.immuni.2021.10.012 34758337

[12] Efron PA Martins A Minnich D Tinsley K Ungaro R Bahjat FR. Characterization of the systemic loss of dendritic cells in murine lymph nodes during polymicrobial sepsis. J Immunol. (2004) 173:3035–43. doi: 10.4049/jimmunol.173.5.3035 15322163

[13] Ding Y Chung CS Newton S Chen Y Carlton S Albina JE. Polymicrobial sepsis induces divergent effects on splenic and peritoneal dendritic cell function in mice. Shock. (2004) 22:137–44. doi: 10.1097/01.shk.0000131194.80038.3f, PMID: 15257086 PMC2253681

[14] Kang SJ Liang HE Reizis B Locksley RM. Regulation of hierarchical clustering and activation of innate immune cells by dendritic cells. Immunity. (2008) 29:819–33. doi: 10.1016/j.immuni.2008.09.017, PMID: 19006696 PMC2858430

[15] Roquilly A McWilliam HEG Jacqueline C Tian Z Cinotti R Rimbert M. Local modulation of antigen-presenting cell development after resolution of pneumonia induces long-term susceptibility to secondary infections. Immunity. (2017) 47:135–147.e5. doi: 10.1016/j.immuni.2017.06.021, PMID: 28723546

[16] Granjeaud S Naquet P Galland F. An ESTs description of the new Vanin gene family conserved from fly to human. Immunogenetics. (1999) 49:964–72. doi: 10.1007/s002510050580, PMID: 10501839

[17] Martin F Malergue F Pitari G Philippe JM Philips S Chabret C. Vanin genes are clustered (human 6q22-24 and mouse 10A2B1) and encode isoforms of pantetheinase ectoenzymes. Immunogenetics. (2001) 53:296–306. doi: 10.1007/s002510100327, PMID: 11491533

[18] Dupre S Cavallini D. Purification and properties of pantetheinase from horse kidney. Methods Enzymol. (1979) 62:262–7. doi: 10.1016/0076-6879(79)62227-9 440106

[19] Kavian N Mehlal S Marut W Servettaz A Giessner C Bourges C. Imbalance of the Vanin-1 pathway in systemic sclerosis. J Immunol. (2016) 197:3326–35. doi: 10.4049/jimmunol.1502511, PMID: 27647831

[20] Penet MF Abou-Hamdan M Coltel N Cornille E Grau GE de Reggi M. Protection against cerebral malaria by the low-molecular-weight thiol pantethine. Proc Natl Acad Sci U S A. (2008) 105:1321–6. doi: 10.1073/pnas.0706867105, PMID: 18195363 PMC2234136

[21] Prisco D Rogasi PG Matucci M Paniccia R Abbate R Gensini GF. Effect of oral treatment with pantethine on platelet and plasma phospholipids in IIa hyperlipoproteinemia. Angiology. (1987) 38:241–7. doi: 10.1177/000331978703800307, PMID: 3551695

[22] Ling L Lu HT Wang HF Shen MJ Zhang HB. MicroRNA-203 acts as a potent suppressor in septic shock by alleviating lung injury via inhibition of VNN1. Kidney Blood Press Res. (2019) 44:565–82. doi: 10.1159/000500484, PMID: 31340209

[23] Fu JD Gao CH Li SW Tian Y Li SC Wei YE. Atractylenolide III alleviates sepsis-mediated lung injury via inhibition of FoxO1 and VNN1 protein. Acta Cir Bras. (2021) 36:e360802. doi: 10.1590/acb360802, PMID: 34644770 PMC8516425

[24] Gudiol C Albasanz-Puig A Cuervo G Carratalà J. Understanding and managing Sepsis in patients with Cancer in the era of antimicrobial resistance. Front Med (Lausanne). (2021) 8:636547. doi: 10.3389/fmed.2021.636547 33869250 PMC8044357

[25] Chai CY Zhang Y Song J Lin SC Sun S Chang IW. VNN1 overexpression is associated with poor response to preoperative chemoradiotherapy and adverse prognosis in patients with rectal cancers. Am J Transl Res. (2016) 8:4455–63. PMID: 27830030 PMC5095339

[26] Kang M Qin W Buya M Dong X Zheng W Lu W. VNN1, a potential biomarker for pancreatic cancer-associated new-onset diabetes, aggravates paraneoplastic islet dysfunction by increasing oxidative stress. Cancer Lett. (2016) 373:241–50. doi: 10.1016/j.canlet.2015.12.031 26845448

